# *Clostridium butyricum* improves the intestinal health of goats by regulating the intestinal microbial community

**DOI:** 10.3389/fmicb.2022.991266

**Published:** 2022-09-20

**Authors:** Chengrui Zhang, Tingyi Hou, Qingyuan Yu, Jihong Wang, Miao Ni, Yunfei Zi, Hangshu Xin, Yonggen Zhang, Yukun Sun

**Affiliations:** ^1^College of Animal Science and Technology, Northeast Agricultural University, Harbin, China; ^2^Ordos Academy of Agriculture and Animal Husbandry, Ordos, China

**Keywords:** *Clostridium butyricum*, intestine, epithelial barrier, microbiota, goats

## Abstract

*Clostridium butyricum*, as a probiotic with a variety of active products, has been widely used to improve the intestinal health of humans and animals. Previous studies had demonstrated that *Clostridium butyricum* exhibited potential protective and positive effects in human disease research and animal production by producing a variety of beneficial substances, such as intestinal inflammation, the intestinal epithelial barrier, metabolic diseases, and regulation of the gut microbiota. Therefore, we hypothesized that dietary *Clostridium butyricum* supplementation could improve gut health in fattening goats by modulating gut microbiota. However, it is unclear whether *Clostridium butyricum* can reach the intestine through the rumen, so 15 healthy Albas goats were selected and randomly divided into 3 treatments with 5 replicates in each group. The groups were divided as follows: control group (CON: basal diet), rumen-protected *Clostridium butyricum* group (RPCB: basal diet plus 1.0 × 10^9^ CFU/kg *Clostridium butyricum* coated with hydrogenated fat), and *Clostridium butyricum* group (CB: basal diet plus 1.0 × 10^9^ CFU/kg *Clostridium butyricum*). The experiment was slaughtered after a 70-day growth test, and the jejunal mucosa and intestinal contents of the goats were collected to determine tight junction proteins related genes expression and 16S rDNA microbial sequencing analysis to evaluate the intestine health. The results showed that dietary supplementation with *Clostridium butyricum* significantly increased the expression of the *Claudin-4* gene of the jejunal mucosa (*P* < 0.05) and had a trend toward a significant increase in the *Occludin* gene (0.05 < *P* < 0.10). However, *Clostridium butyricum* had no significant effect on the expression of intestinal inflammatory factors (*P* > 0.10). In addition, the relative fractionation of *Clostridium* and *Clostridiaceae_unclassified* in the gut microbiota at the genus level decreased significantly compared with controls (*P* < 0.05). The results of the analysis of the level of *Clostridium* species showed that *Clostridium butyricum* only existed in the treatment group. And the correlation results showed that *Occludin* and *Claudin-4* genes were positively correlated with *Sharppea* and *Clostridium butyricum*, and negatively correlated with *Clostridium* (*P* < *0.05*). Supplementing *Clostridium butyricum* in the diet did not significantly affect the intestinal immune function of goats, while regulation of the intestinal microbiota was associated with improving the intestinal epithelial barrier.

## Introduction

Intensive and grazing farming are the main way of feeding goats and sheep in China (Mu et al., 2020), among which high-concentrate fattening is a common production method to meet their energy needs in the short term to improve growth performance (Wang M. et al., 2021), which undoubtedly aggravates the immune pressure and gastrointestinal metabolic load of ruminants (Plaizier et al., 2018). Due to the unique digestive systems of ruminants, many major studies have focused on the effects of high-concentrate diets on rumen fermentation, ruminal acidosis, and their microbial properties and functions. It is well known that the small intestine is the main place for nutrient absorption, and the intestine is closely related to its autoimmune function as the largest immune organ in the body (Chelakkot et al., 2018; Mu et al., 2021). Therefore, paying attention to the intestinal health of goats and sheep during the rapid fattening stage has important implications for their production.

Diarrhea is one of the important reasons affecting the growth and production performance of goats and sheep during intensive and grazing farming (Wang M. et al., 2021). A large number of studies have shown that the impaired intestinal epithelial barrier (Ma et al., 2020), low functional immunity of the gastrointestinal tract (Peng et al., 2021), and intestinal flora disturbance can cause diarrhea of different degrees (Zhang et al., 2021). Diarrhea affects the body’s absorption of small molecular substances and nutritional elements (Esterházy et al., 2019), destroys intestinal homeostasis, and results in severe loss of body fluids (McLamb et al., 2013). The intestinal epithelial barrier is an important line of defense in the body to exert immune function by improving the expression of intestinal epithelial tight junction (TJ) proteins, intestinal epithelial cells’ development, and the mucus layer’s physiological characteristics. Strengthening the intestinal epithelial barrier function can help inhibit intestinal inflammation and the occurrence of diarrhea caused by various factors (Chelakkot et al., 2018). Dietary supplementation with natural active substances (such as plant essential oils, probiotics, and peptides) is a common production method to improve animal gut health and positively impacts growth performance, antioxidant levels, and rumen fermentation (Xie et al., 2020, 2021; Hagihara et al., 2021).

*Clostridium butyricum* (*C. butyricum*) is a class of anaerobic probiotics with spores that are resistant to bile salts and gastric acid, and have strong resistance to stress (Hagihara et al., 2020). Its rich metabolites such as butyric acid (Stoeva et al., 2021), digestive enzymes (Zhan et al., 2019), bacteriocin (Wang T. et al., 2021), vitamins, and other nutrients (Araki et al., 2002) have a variety of probiotic functions, including enhancement of intestinal epithelial barrier integrity, anti-inflammatory and modulation of intestinal flora. Previous studies have demonstrated that supplementation with *C. butyricum* improves gut health and nutrient absorption of humans and livestock (Zhang et al., 2018; Wang et al., 2020; Stoeva et al., 2021). Meanwhile, dietary supplementation with butyrate has also been reported to enhance the regulation of intestinal epithelial barrier, inflammation, and microflora (Bedford and Gong, 2018). However, the regulation of intestinal health and function by *C. butyricum* in ruminants is rarely reported.

Overall, we hypothesized that *C. butyricum* could reach the goat intestinal tract through the rumen and had a positive effect. Therefore, the purpose of this experiment was to compare the impact of different forms of *C. butyricum* on the jejunal TJ proteins expression, anti-inflammatory level and intestinal flora of goats at the same supplementation level, and to evaluate the regulation of *C. butyricum* on intestinal health of goats.

## Materials and methods

The current experimental protocol was approved by the Institutional Animal Care and Use Committee of Northeast Agricultural University (Harbin, China) (protocol number: NEAU- [2011]-9).

### Experimental design and animal management

Fifteen good health Albas male goats with 21.9 ± 0.1 kg of body weight (BW) were used. In a randomized trial design, these goats were divided into three treatments (5 goats per treatment). The control (CON) goats were fed a basal diet, rumen-protected *C. butyricum* (RPCB), and *C. butyricum* (CB) goats were fed a basal diet with 1 × 10^9^ CFU/kg *C. butyricum* (*C. butyricum* was provided by Lvxue Biotechnology Co., Ltd., Hubei, China); having effective colony count ≥ 1 × 10^9^ CFU/g. Rumen-protected *C. butyricum* was protected by hydrogenated fat, and the effective colony count was the same as *C. butyricum*. All goats were fed with the concentrate to forage ratio of the experimental diet of 65:35 (forage is a mixture of oat and alfalfa; concentrate was provided by Commercial concentrate Jiuzhou earth feed Co., Ltd. without probiotics, Inner Mongolia, China). Each treatment was fed for 14 days of adaptation, and then all goats were fed for 56 days. Goats were fed twice daily at 06:00 and 18:00. Each goat was arranged in a single pen with free access to fresh water. The ingredient and nutrient composition of diets are shown in Table 1.

**TABLE 1:** Composition and nutrient levels of diets (DM basis, %).

Ingredients	Contents	
	Concentrate	Mixed forages
Corn	40	
Corn germ meal	20	
Shotcrete corn husk	13	
DDGS	10	
Extruded soybean	8	
Molasses	3	
Limestone	4	
NaCl	1	
Compound premix^a^	1	
Total	100	
Nutrient levels^b^		
Dry matter	90.38	92.04
Crude protein	18.93	11.67
Ether extract	4.72	2.15
Crude ash	6.02	7.69
Neutral detergent fiber (NDF)	17.58	55.75
Acid detergent fiber (ADF)	6.01	35.39
Metabolizable energy (MJ/kg)	14.09	14.05

^a^Each kilogram of composite premix includes: Ca 1.54 g, P 0.51 g, Fe 25 mg, Zn 35 mg, Cu 8 mg, Co 0.1 mg, I 0.9 mg, Se 0.25 mg, Mn 19.5 mg, VE 1000 IU, VA 3000 IU, VD 1000 IU.

^b^ME was a calculated value, while the others were measured values.

### Sample collection

After the growth trial, all experimental goats were electrically stunned (200 V applied for 4 s) and slaughtered by exsanguination. Then, the jejunum was collected and washed immediately with PBS buffer. And then, the collected jejunum samples were divided into two parts. The mucosa was scraped off the jejunum with sterile slides and placed in a cryopreservation tube immediately after being frozen in liquid nitrogen and stored in a −80°C refrigerator to be transferred to the laboratory for evaluating the expression of TJ proteins and inflammatory factor. Meanwhile, the contents of the intestine were placed in presterilized cryovials, frozen in liquid nitrogen, and then transferred to the laboratory’s −80°C refrigerator for storage to measure microbial diversity.

### Quantitative real-time PCR

Total RNA was extracted from the jejunal mucosa of each goat using an animal tissue total RNA extraction kit (TIANGEN Bio, Beijing, China). Ultra-Micro Spectrophotometer (Implen P330, Munich, Germany) was used to determine RNA concentration and quality. The absorbance ratio of all samples was between 1.8 and 2.2, indicating the high quality of the total RNA extracted. Subsequently, it was reverse transcribed into first-strand cDNA according to the 5X Integrated RT MasterMix kit (DiNing Bio, Beijing, China). The relevant gene expression primers (Table 2) used in this experiment were designed and synthesized by General Biological Co., Ltd. Real-time quantitative PCR was performed using the 2x Fast qPCR Master Mixture kit (DiNing Bio, Beijing, China) with the Quantagene q225MX Real-Time PCR System (KuBo Technology, Beijing, China) according to the manufacturer’s instructions. Each sample was analyzed in triplicate, the mean value of each target gene and β-actin’s *Ct*-values were calculated, and ΔCt was determined. The 2^–ΔΔ*Ct*^ method was used to analyze the relative expression of each target gene.

**TABLE 2:** Primer sequences, genes targeted and length of quantitative real-time PCR products.

Target genes	GenBank accession no.	Primer sequences^a^	Product (bp)
*ACTB*	NM_001314342.1	F: GGCTACAGCTTCACCACCAC R: GGAAGGAAGGCTGGAAGAGAG	211
*Occludin*	XM_018065681.1	F: AGCAGCAGCGGTAACTTGG R: CGTCGTGTAGTCTGTTTCATAGTGG	108
*Claudin-1*	XM_005675123.3	F: ACAGCACTCTGCAAGCAACC R: TTCTGTGCCTCGTCGTCTTC	124
*Claudin-4*	XM_005697785.2	F: CCGCCACGAAACAACAAG R: GGGAGAAACAAAGACGAAAGGA	129
*ZO-1*	XM_018066118.1	F: CCGAATGAAACCACACACAAA R: TCCACGCCACTGTCAAACTC	104
*TNF*-α	NM 001286442.1	F: CCACTGACGGGCTTTACCT R: TGATGGCAGAGAGGATGTTG	141
*IL-1*β	DQ837160.1	F: AAGGCTCTCCACCTCCTCTC R: TTGTCCCTGATACCCAAGG	114
*IL-6*	NM 001285640.1	F: TGACTTCTGCTTTCCCTACCC R: GCCAGTGTCTCCTTGCTGTT	193
*IL-10*	XM_005690416.3	F: GTGATGCCACAGGCTGAGAAC R: GAAGATGTCAAACTCACTCATGG	213

^a^F, forward; R, reverse.

### Microbial composition

DNA from all contents of cecum samples was extracted using the E.Z.N.A.^®^ Stool DNA Kit (D4015, Omega, Inc., USA) according to the manufacturer’s instructions. Nuclear-free water was used for blank. The total DNA was eluted in 50 μL of elution buffer and stored at −80°C until measurement using PCR by LC-Bio Technology Co., Ltd. (Zhejiang, China). Universal primer sequences were designed according to the V3-V4 region of the amplified fragment of 16S rDNA: 341F (5′-CCTACGGGNGGCWGCAG-3′, 805R (5′-GACTACHVGGGTATCTAATCC-3′), each sample barcoded at the 5′ end of the primers, and sequenced the universal primers. PCR amplification was performed in a total volume of 25 μL of reaction mix containing 25 ng of template DNA, 12.5 μL of PCR master mix, 2.5 μL of each primer, and PCR-grade water was added to adjust the volume. PCR conditions for amplifying prokaryotic 16S fragments: initial denaturation at 98°C for 30 s; denaturation at 98°C for 10 s, annealing at 54°C for 30 s, extension at 72°C for 45 s, 32 cycles; and final extension at 72°C for 10 min. Post-PCR products were confirmed by 2% agarose gel electrophoresis. During the entire DNA extraction process, ultrapure water was used instead of sample solution to exclude the possibility of false-positive PCR results as negative controls. Beads (Beckman Coulter Genomics, Danvers, MA, USA) were purified and quantified by Qubit (Invitrogen, USA). Amplicon pools were used for sequencing, and the size and number of amplicon libraries were measured on an Agilent 2100 Bioanalyzer (Agilent, USA) and Illumina (Kapa Biosciences, Woburn, MA, USA) library quantification kits. Libraries were sequenced on the NovaSeq PE250 platform.

### Statistical analysis

Data were analyzed using the one-way ANOVA process in SPSS 25 statistical software (SPSS 20.0; SPSS Inc., Chicago, IL, USA) followed by Duncan’s multiple range tests, and the test results were expressed as the mean and standard error of the mean (SEM), where *P* < 0.05 was considered statistically significant, and 0.05 < *P* < 0.10 was considered to have a significant trend (Zhou et al., 2019).

16S rDNA sequencing samples were sequenced on the Illumina NovaSeq platform following the manufacturer’s recommendations provided by LC-Bio. Paired-end sequences were assigned to samples based on their unique barcodes, and the barcodes and primer sequences introduced by the library were removed. FLASH was used to merge matching end reads. According to fqtrim (v0.94), the raw read data is quality filtered under specific filtering conditions to obtain high-quality clean labels. Chimeric sequences were filtered using Vsearch software (v2.3.4). DADA2 was used to demodulate to get the feature table and feature sequence. Alpha diversity and beta diversity are calculated by normalizing to the same random sequence. Feature abundances were then normalized using the relative abundance of each sample according to the SILVA (release132) classifier. Alpha diversity is used to analyze the complexity of sample species diversity through 5 metrics, including Chao, Observed species, Goods coverage, Shannon, Simpson, all of which are calculated with QIIME2 in our sample. Beta diversity was calculated by QIIME2 and plotted by the R package. Both the correlation network graph and the correlation heatmap are analyzed through the Spearman correlation matrix, which is also drawn by the R package (v3.6.3). The BLAST was used for sequence alignment, and each representative sequence was annotated with the SILVA database for characteristic sequences. Other graphs are also implemented using the R package (v3.5.2).

## Results

### Gene expression of tight junction proteins in the jejunal mucosa

Table 3 shows the preliminary analysis results of β*-actin* (*ACTB*), *Occludin*, *Claudin-1*, *Claudin-4*, and *Zonula occludens-1* (*Zo-1*) mRNA expression levels in fattening goats. The mRNA expression of *Claudin-4* in the jejunal mucosa significantly increased in CB treatment (*P* < 0.05). In addition, the mRNA expression of *Occludin i*n CB had an upward trend compared to the CON (*P* < 0.05). However, the expression of *ACTB*, *Claudin-1*, and *ZO-1* were not markedly different in the treatment group (*P* > 0.10).

**TABLE 3:** The mRNA expression of TJ proteins of the jejunal mucosa of goats with diet supplementation with *C. butyricum*.

	Treatments^1^				
Items	CON	RPCB	CB	SEM^2^	*P*-value
*ACTB*	0.99	1.16	1.02	0.10	0.795
*Occludin*	0.68^b^	1.14^ab^	1.73^a^	0.19	0.055
*Claudin-1*	2.01	2.52	1.79	0.60	0.892
*Claudin-4*	0.73^b^	1.08^ab^	1.46^a^	0.12	0.026
*Zo-1*	0.65	1.59	1.77	0.32	0.344

^1^Treatments: CON, control group without on the basic diet; RPCB, ad 1.0 × 10^9^ CFU rumen protected *C. butyricum* per kg of basic diet, DM basis; CB, ad 1.0 × 10^9^ CFU *C. butyricum* per kg of basic diet, DM basis.

^2^SEM: Standard error of the means (*n* = 5 goats/group).

^ab^Mean values with different superscripts are significantly different (*P* < 0.05).

### Gene expression of inflammatory factors in the jejunal

The results of the expression of *TNF*-α, *IL-1*β, *IL-6*, and *IL-10* in the colon of fattening goats are shown in Table 4. The mRNA expressions of *TNF*-α, *IL-1*β, *IL-6*, and *IL-10* in the colon were without significant differences in the above treatment groups (*P* > 0.10).

**TABLE 4:** The mRNA expression of inflammatory factors of the jejunal of goats with diet supplementation with *C. butyricum*.

	Treatments^a^				
Items	CON	RPCB	CB	SEM^b^	*P*-value
*TNF*-α	0.87	1.00	2.03	0.34	0.335
*IL-1*β	1.31	0.89	1.84	0.34	0.554
*IL-6*	0.97	2.19	2.12	0.48	0.549
*IL-10*	0.57	1.83	2.64	0.55	0.326

^a^Treatments: CON, control group without on the basic diet; RPCB, ad 1.0 × 10^9^ CFU rumen protected *C. butyricum* per kg of basic diet, DM basis; CB, ad 1.0 × 10^9^ CFU *C. butyricum* per kg of basic diet, DM basis.

^b^SEM: Standard error of the means (*n* = 5 goats/group).

### The content of cecum microbial composition

**FIGURE 1:**
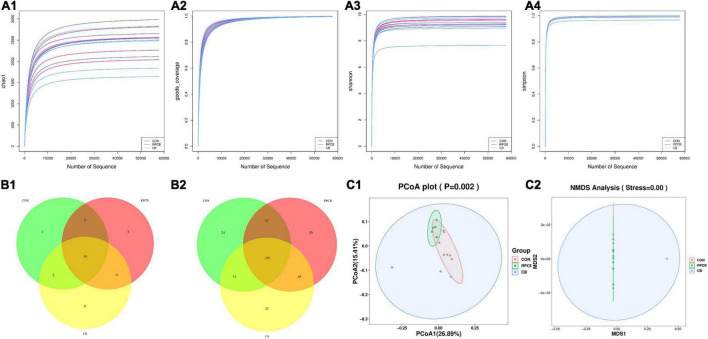
The α- and β-diversity of fecal bacterial communities in fattening goats fed *C. butyricum*. Chao 1 **(A1)**, Goods coverage **(A2)**, Shannon **(A3)**, and Simpson **(A4)** curves of intestine microbiome of fattening goats are shown. Unique and shared intestine feature among CON, RPCB and CB groups shown on Venn diagrams in phylum **(B1)** and genus **(B2)** level. Intestine microbial structure among the three groups was estimated by the Principal Coordinate Analysis **(C1)** and Non-metric Multidimensional Scaling Analysis **(C2)**.

A total of 1,098,998 effective 16S rDNA sequences were detected in the content of 15 cecum samples. After filtering (proportion of data with data quality ≥ Q20 and Q30 in valid data), the values of all samples were better than 98.2 and 97.4% indicating good sequencing depth for analysis of the content of cecum microbiota. The amount of data measured is within a reasonable range, as shown by the dilution curve in α-diversity (Figures 1A1–A4), but there is no significant difference between them. Common and specific bacteria at the phylum and genus levels are shown in the Venn diagrams (Figures 1B1,B2). The microbiota of the three treatments shared 25 phyla and 274 genera. However, the PCoA and NMDS plots analysis are one of the most commonly used analytical methods of β-diversity performed, and the compositions of cecum microbial were statistically different among the above three treatments (*P* < *0.05*) (Figures 1C1,C2).

**FIGURE 2:**
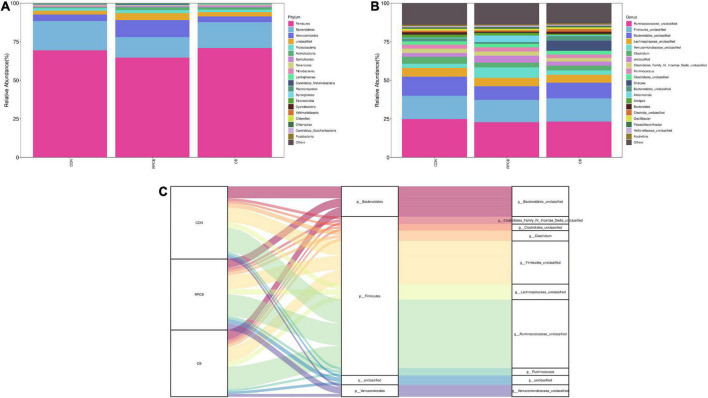
Stacked bar graph displays a comparison of microbial relative abundance (%) at phylum **(A)** and genus **(B)** level among main effects of treatments in intestine of fattening goats. And Sankey plot displays a comparison of microbial relative abundance (%) at genus level **(C)** among main effects of treatments in intestine of fattening goats. The label CON denotes without prebiotics, the label RPCB denotes rumen-protected *C. butyricum*, and the label CB denotes *C. butyricum* for each sample. CON: 0 CFU *C. butyricum* per kilogram feed, RPCB: 1.0 × 10^9^CFU *C. butyricum* per kilogram feed, CB: 1.0 × 10^9^CFU *C. butyricum* per kilogram feed.

In addition, we analyzed phylum- and genus-level microbiomes to assess their specific composition (Tables 5, 6). The relative abundances of different treatments at different levels are represented by histograms (Figures 2A,B). Bacterial taxa with a relative abundance > 0.1% in more than 3 biological replicates per treatment were considered as identified in the current study. According to the result of phylum, we found that Firmicutes predominated in each treatment. Furthermore, the relative abundance of Verrucomicrobia and unclassified in the RPCB group was significantly higher than in the CON and CB groups (*P* < 0.05). In addition, *Candidatus_Melainabacteria* had an increasing trend in the RPCB (0.05 < *P* < 0.1). The rest did not change significantly. At the genus level, the relative abundance of *Clostridium*, *Clostridiaceae_unclassified*, and *Parapedobacter* were markedly decreased in RPCB and CB compared with CON (*P* < 0.05). The relative abundance of R*ikenellaceae _unclassified* and *unclassified* was significantly increased only in RPCB. Meanwhile, the relative abundances of *Akkermansia*, *Clostridia_unclassified*, *Candidatus_Melainabacteria_unclassified*, and *Sporobacter* tended to increase compared to the control group (0.05 < *P* < 0.1). Contrary to this result are *Emticicia* and *Mollicutes_ unclassified* (0.05 < *P* < 0.1). Simultaneously, the Sankey plot shows that the common core bacteria represented mainly came from the Bacteroidetes, Firmicutes, Verrucomicrobia, and unclassified phylum (Figure 2C). Moreover, we also further explored the species level of *Clostridium* (Table 7). As indicated in the results, the relative fractions of some uncultured and unclassified *Clostridium* species were significantly reduced (*P* < 0.05) or tended to decline (0.05 < *P* < 0.1). There was no significant change in the relative abundance of *C. butyricum* in each group, and it only existed in RPCB and CB treatments.

**TABLE 5:** Effects of *C. butyricum* on the relative abundance (> 0.1%) of gut microbes at the phylum level in goats.

	Treatments^1^				
Items	CON	RPCB	CB	SEM^2^	*P*-value
Firmicutes	69.43	64.65	70.86	1.92	0.414
Bacteroidetes	18.99	13.34	16.84	1.63	0.391
Verrucomicrobia	4.20^b^	11.00^a^	3.68^b^	1.22	0.010
Unclassified	2.45^b^	4.58^a^	2.70^b^	0.38	0.030
Proteobacteria	1.69	1.96	1.67	0.15	0.698
Actinobacteria	0.54	1.94	1.55	0.52	0.565
Sirochaetes	0.72	0.77	1.11	0.13	0.469
Tenericutes	0.39	0.29	0.39	0.07	0.828
Fibrobacteres	0.48	0.20	0.22	0.10	0.463
Lentishaerae	0.28	0.29	0.24	0.05	0.922
Candidatus Melainabacteria	0.22	0.41	0.12	0.06	0.099
Lanctomycetes	0.21	0.15	0.21	0.03	0.761
Synergistetes	0.17	0.13	0.22	0.06	0.871
Elusimicrobia	0.08	0.14	0.05	0.02	0.315

^1^Treatments: CON, control group without on the basic diet; RPCB, ad 1.0 × 10^9^ CFU rumen protected *C. butyricum* per kg of basic diet, DM basis; CB, ad 1.0 × 10^9^ CFU *C. butyricum* per kg of basic diet, DM basis.

^2^SEM: Standard error of the means (*n* = 5 goats/group).

^ab^Mean values with different superscripts are significantly different (*P* < 0.05).

**TABLE 6:** Effects of *C. butyricum* on the relative abundance (> 0.1%) of gut microbes at the genus level in goats.

	Treatments^1^				
Items	CON	RPCB	CB	SEM^2^	*P*-value
*Ruminococcaceae_unclassified*	24.81	22.80	23.22	1.13	0.774
*Firmicutes_unclassified*	15.19	14.53	15.02	0.73	0.939
*Bacteroidales_unclassified*	12.34	8.81	10.16	1.24	0.538
*Lachnospiraceae_unclassified*	5.63	5.38	5.14	0.47	0.925
*Verrucomicrobiaceae_unclassified*	2.67	6.78	2.88	1.04	0.196
*Clostridium*	4.58^a^	3.08^b^	3.00^b^	0.30	0.042
*Unclassified*	2.45^b^	4.58^a^	2.70^b^	0.38	0.030
*Clostridiales_Family_IV._Incertae_Sedis_unclassified*	2.75	2.70	2.29	0.30	0.816
*Ruminococcus*	2.62	2.74	2.26	0.33	0.845
*Clostridiales_unclassified*	2.23	2.17	2.42	0.20	0.883
*Sharpea*	0.00^b^	0.04^ab^	0.13^a^	0.02	0.098
*Bacteroidetes_unclassified*	1.90	1.24	2.89	0.37	0.186
*Akkermansia*	0.24^b^	4.15^a^	0.58^ab^	0.80	0.068
*Alistipes*	1.92	1.78	0.88	0.31	0.363
*Bacteroides*	1.59	0.75	1.49	0.23	0.287
*Clostridia_unclassified*	1.00^b^	0.81^b^	1.64^a^	0.16	0.086
*Oscillibacter*	1.23	1.01	1.15	0.11	0.718
*Pseudoflavonifractor*	0.93	1.22	0.97	0.09	0.404
*Veillonellaceae_unclassified*	0.74	0.95	0.71	0.06	0.256
*Acetivibrio*	0.87	0.66	0.85	0.11	0.714
*Treponema*	0.65	0.61	1.08	0.12	0.241
*Olsenella*	0.12	1.41	0.75	0.46	0.562
*Clostridiaceae_unclassified*	0.71^a^	0.24^b^	0.17^b^	0.07	0.000
*Candidatus_Melainabacteria_unclassified*	0.22^ab^	0.41^a^	0.12^b^	0.06	0.099
*Bifidobacterium*	0.13	0.13	0.43	0.07	0.085
*Others*	9.79	8.61	8.56	0.46	0.504

^1^Treatments: CON, control group without on the basic diet; RPCB, ad 1.0 × 10^9^ CFU rumen protected *C. butyricum* per kg of basic diet, DM basis; CB, ad 1.0 × 10^9^ CFU *C. butyricum* per kg of basic diet, DM basis.

^2^SEM: Standard error of the means (*n* = 5 goats/group).

^ab^Mean values with different superscripts are significantly different (*P* < 0.05).

**TABLE 7:** Effects of *C. butyricum* on the relative abundance (> 0.1%) of gut microbes at the species level (*Clostridium* species) in goats.

	Treatments^1^				
Items	CON	RPCB	CB	SEM^2^	*P*-value
*Uncultured_Clostridium_sp.*	4.39^a^	2.82^b^	2.61^b^	0.306	0.020
*Clostridiales_Family_IV._Incertae_Sedis_unclassified*	2.75	2.70	2.29	0.303	0.816
*Clostridiales_unclassified*	2.23	2.17	2.42	0.202	0.883
*Clostridia_unclassified*	1.00^ab^	0.81^b^	1.64^a^	0.164	0.086
*Clostridiaceae_unclassified*	0.71^a^	0.24^b^	0.17^b^	0.075	0.000
*Clostridiales_Family_XIV._Incertae_Sedis_unclassified*	0.25	0.17	0.26	0.036	0.565
*Clostridiales_Family_XIII._Incertae_Sedis_unclassified*	0.19	0.18	0.16	0.018	0.878
*Erysipelatoclostridium_sp._SNUG30386*	0.15	0.07	0.15	0.023	0.348
*Clostridiumsp.enrichmentc_ulturecloned7*	0.08	0.10	0.11	0.013	0.625
*Clostridiales_Family_XII._Incertae_Sedis_unclassified*	0.07	0.15	0.01	0.032	0.206
*Clostridium_sp._Clone7*	0.04	0.05	0.11	0.022	0.442
*Clostridium butyricum* ^3^	0.00	0.01	0.02	0.005	0.232

^1^Treatments: CON, control group without on the basic diet; RPCB, ad 1.0 × 10^9^ CFU rumen protected *C. butyricum* per kg of basic diet, DM basis; CB, ad 1.0 × 10^9^ CFU *C. butyricum* per kg of basic diet, DM basis.

^2^SEM: Standard error of the means (*n* = 5 goats/group).

^3^*C. butyricum* is a special concern at the *Clostridium* species level.

^ab^Mean values with different superscripts are significantly different (*P* < 0.05).

**FIGURE 3:**
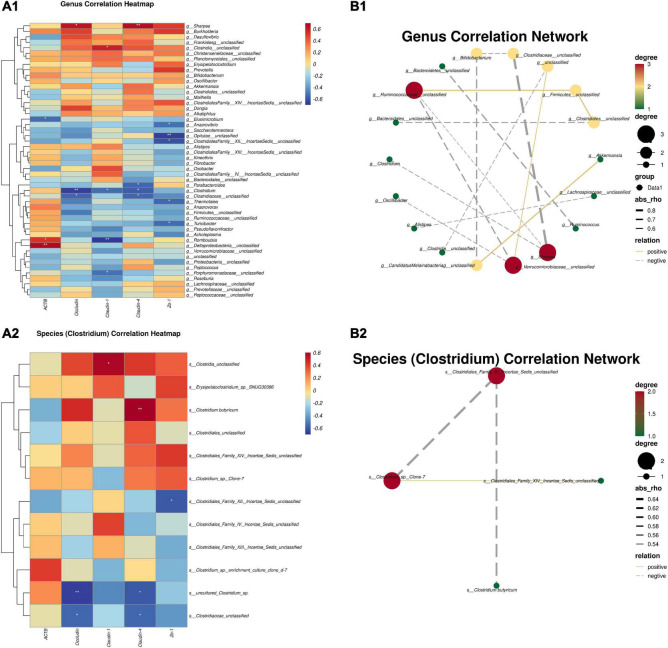
Spearman correlation between intestinal microbiotas and the mRNAs TJ proteins. **(A1)** Genus correlation heatmap; **(A2)** species (*Clostridium*) correlation heatmap; **(B1)** genus correlation network; **(B2)** species (*Clostridium*) correlation network. Red denotes a positive correlation; blue denotes a negative correlation. The color intensity is proportional to the strength of the Spearman correlation. *P ≤ 0.05, **P ≤ 0.01.

According to the Spearman correlation algorithm, we performed a correlation analysis of gut microbes and mRNAs related to the jejunal mucosal barrier. As the heatmap shows, the mRNA expressions of *Occludin* and *Claudin-4* performed a positive correlation with the Sharpea genus (*P* < 0.05) (Figure 3A1). In addition, *Claudin-4* was strongly positively correlated with *C. butyricum* (*P* < 0.01), and *Occludin* showed a positive correlation with *C. butyricum* (red in the correlation heatmap) in the species correlation heatmap (Figure 3A2). This suggests that *C. butyricum* could positively affect the expression of TJ proteins in jejunal mucosal. Meanwhile, we also correlated microbes at the genus and species level (*Clostridium*). The results in Figure 3 show interactions between core bacteria at different levels through the correlation analysis of the core microbiomes at the genus level and the microbiomes with significant and significant trends. Among them, the centralities of *Ruminococaceae_unclassified*, *Verrucomicrobiaceae_unclassified*, and *sharpea* were the largest, while the centralities of *Bifidobacterium*, *Clodtridiaceae_unclassified*, *unclassified*, *Firmicutes_unclassified*, and *Clostridales_unclassified* were in the middle, and the centralities of the rest of the microbiomes were the lowest. Of particular concern, *Clostridium* was only negatively associated with *Sharpea* and not with other microbiomes (Figure 3B1). In addition, through the significant species (*Clostridium*) correlation network analysis, only the *Clostridiales_Family_IV._Incertae_Sedis-_unclassified* and *Closrtidium_*sp._*Clone-7* were the largest core microbiomes, of which *C. butyricum* was negatively correlated with *Clostridiales_Family_IV._Incertae_Sedis-_unclassified* (Figure 3B2).

## Discussion

The current study aimed to describe how dietary supplementation with *C. butyricium* affects the intestinal tract’s health by adapting the goats’ microbial community. Our previous research indicated that different doses of *C. butyricium* could modulate beneficial microbial diversity and relative abundance in the rumen and feces of fattening goats (Zhang C. et al., 2022).

The efficient digestion, absorption, and transport of nutrients in the gastrointestinal tract is inseparable from the strong immune function of the intestinal tract, which provides a good guarantee for its metabolism in the body (Penner et al., 2011). Previous studies reported that probiotics such as *Bifidobacterium* and *Lactobacillus* colonized the intestinal mucosa of newborn ruminants, inhibited the invasion of harmful bacteria into the intestine, reduced diarrhea in young ruminants, and maintained the homeostasis of the intestinal environment to improve its growth performance (Zhang et al., 2021). In recent years, most studies have reported that *C. butyricum* could regulate the growth performance of animals by balancing the intestinal micro ecological environment and protecting intestinal epithelial cells through its probiotic metabolites (Wang et al., 2020; Meng et al., 2021; Xu et al., 2021). Cytokines play an important role in improving intestinal immune function. The study by Rakhshandeh and de Lange (2012) reported that pro/anti-inflammatory factors were cytokines released after pathogenic bacteria invaded the body, which was closely related to various metabolic reactions, including changing the digestion and absorption of nutrients to improve immune function. Studies in piglets (Liang et al., 2020), poultry (Xu et al., 2021), mice (Hagihara et al., 2020), and aquaculture (Meng et al., 2021) confirmed that dietary supplementation with *C. butyricum* could alter anti-inflammatory factors (*IL-10*) and pro-inflammatory factors (*TNF*-α, *IL-1*β, and *IL-6*, etc.) expressions. However, it was found in this experiment that supplementation with different forms of *C. butyricum* had no significant effect on the expression of *TNF*-α, *IL-1*β, *IL-6*, and *IL-10* in the intestinal tract of goats. This may be related to our previous study that supplementation with *C. butyricum* failed to alter nutrient metabolism in goats, resulting in no significant changes in daily weight gain and feed efficiency (Zhang C. et al., 2022).

While interestingly, the mRNA expressions of TJ proteins *Occludin* and *Claudin-4* in the jejunal mucosa of goats fed a diet containing *C. butyricum* showed a significant upward trend and were significantly increased. As an important part of the intestinal barrier, TJ proteins are a multi-molecular complex composed of various transmembrane proteins, including *ACTB*, *ZO-1, Occludin*, and *Claudin* family proteins (Ulluwishewa et al., 2011). The interaction maintains the paracellular permeability, consolidating the intestinal epithelial barrier function and preventing the penetration of antigenic substances such as lipopolysaccharide from harmful bacteria into the intestinal lamina propria, triggering an inflammatory response (Wageha et al., 2017; Stoeva et al., 2021). The results of this study are consistent with the results of Xu et al. (2021) and Li et al. (2021)’s supplementation of *C. butyricum* could increase the expression of *Occludin* and *Claudin* family genes in the intestinal epithelial barrier. Meanwhile, this might explain the lack of significant changes in the expression of jejunal inflammatory factors after *C. butyricum* supplementation. In addition, Lu et al. (2020) also confirmed in the study of piglets that *C. butyricum* could significantly upregulate the expression of TJ proteins related genes (*ZO-1* and *Occludin*) in the intestinal epithelial barrier. This was inextricably linked to butyric acid, the main metabolite of *C. butyricum*. Most studies have shown that butyric acid has a supporting and protective effect on intestinal epithelial cells, which could provide energy for intestinal epithelial cells and contribute to the growth, development, and improvement of function (Hou et al., 2014; Qiao et al., 2015). Zhao et al. (2020) found that oral administration of *C. butyricum* could improve intestinal mucosal barrier function and reverse intestinal inflammatory damage caused by severe acute pancreatitis and intra-abdominal hypertension by increasing the content of butyric acid and propionic acid in the intestine. In addition, we also learned from Xu et al. (2021) that *C. butyricum* not only altered its metabolites to affect the intestinal epithelial barrier but also consolidated intestinal barrier function by modulating the gut microflora. This also confirmed previous reports that probiotics could help alleviate the decline of TJ proteins expression caused by various reasons (Chang et al., 2019). However, there were no significant differences in other TJ proteins related genes (*ACTB*, *Claudin-1*, and *ZO-1*) in the current study, which were related to the dynamic properties of TJ proteins in the intestinal barrier, the molecular mechanism and its function in different species role related (Xu et al., 2021).

To explore how *C. butyricum* affects the gut epithelial barrier and gut microbes, we performed 16S rDNA sequencing. In the current study, by analyzing the gut microbiota of goats in different groups, the results showed that Firmicutes, Bacteroidetes and Verrucomicrobia were the main dominant microbiota in this intestinal segment. Among them, the dietary supplementation with RPCB significantly increased the relative abundance of Verrucomicrobia. This may be due to the synergy of the unclassified phyla whose relative abundances also rose. Previous studies have shown that Verrucomicrobia can play a beneficial role by improving the body’s metabolism or increasing the thickness of the mucus layer (Everard et al., 2013). It was because butyric acid, as one of the products of *C. butyricum* with other various nutritional products, was positively correlated with the modification of intestinal flora (Zhang Q. et al., 2022). At the genus level, we found significant differences in *Clostridium*, *Unclassified* and *Clostridiaceae_unclassified*. We also found that there were 5 kinds of bacteria with different trends. Surprisingly, supplementation of both forms of *C. butyricum* in goat diets significantly decreased the relative abundance of *C. butyricum* in their intestines. Therefore, we further analyzed the species-level *Clostridium*, and the results showed the relative abundance of *Uncultured_Clostridium_*sp. and *Clostridium_unclassified* in the gut of goats was significantly reduced after supplementation with *C. butyricum*. Because *C. butyricum* was the research object of this experiment, we paid special attention to it. Although its relative abundance in the intestine was not higher than 0.1% and it only existed in the treatment group, while the relative abundance of *C. butyricum* equaled 0% in the control group. It was well known that *Clostridium* also had harmful bacteria detrimental to body health and non-pathogenic microorganisms not classified as competing for nutrients with beneficial bacteria (Amila et al., 2020). Therefore, the significant decrease in the relative abundance of *Clostridium* as well as the mRNA expressions of TJ proteins *ACTB*, *Claudin-1*, and *Zo-1* without obvious difference may be related to the decline of pathogenic *Clostridium* in the gut.

Furthermore, in order to reveal the relationship between the gut microbiota and epithelial barrier function after supplementation with *C. butyricum*, we analyzed the correlation between the gut microbiota and genes related to the expression of TJ proteins in the jejunal mucosa, as well as the correlation between the flora. A previous study had shown that as one of the main producers of lactic acid in the gastrointestinal tract, *Sharpea* could synthesize lactic acid by hydrogenating hydrogen ions in the body through its own lactate dehydrogenase (Sandeep et al., 2018). However, since too much lactic acid cannot be accumulated in the intestinal tract, it can be converted into butyric acid by the action of lactic acid utilizing bacteria (Zhu et al., 2020). In the genus level correlation analysis, *Sharpea* showed a strong positive correlation with the *Occludin* and *Claudin-4* genes that were determined to be significantly expressed. This might be related to the proliferation of *Sharpea* in cooperation with other butyrate-producing bacteria. Moreover, *Clostridium* negatively correlated with *Occludin* and *Claudin-4* genes expression, while species (*Clostridium*) level correlation analysis showed that *Occludin* was positively correlated with *Claudin-4 C. butyricum* and negatively correlated with two unidentified *Clostridium* species. And the *Claudin-4* gene showed a strong correlation with *C. butyricum*. Therefore, this corresponds to our conjecture that supplementation with *C. butyricum* may reduce the harmful bacteria *C. butyricum*, and also means that *C. butyricum* could upregulate the expression of TJ proteins related genes by itself or in coordination with other probiotics (Stoeva et al., 2021).

## Conclusion

Dietary supplementation with *C. butyricum* could enhance the intestinal epithelial barrier function of goats by regulating the relative abundance of beneficial intestinal bacteria, while rumen protection of *C. butyricum* had no significant effect on the intestinal health of goats. Fortunately, this gives us a research direction. In the future, more molecular techniques and bacterial genomics methods should be combined to reveal the specific mechanism of *C. butyricum* regulating the intestinal flora of goats.

## Data availability statement

The datasets presented in this study can be found in online repositories. The names of the repository/repositories and accession number(s) can be found below: NCBI database, the accession number: PRJNA861251.

## Ethics statement

The animal study was reviewed and approved by the Institutional Animal Care and Use Committee of Northeast Agricultural University (Harbin, China). Written informed consent was obtained from the owners for the participation of their animals in this study.

## Author contributions

CZ, YGZ, and YS conceptualized and designed the study. CZ conducted animal trials, analyzed the data, and drafted original manuscript. TH, QY, and JW performed laboratory experiments. YFZ and MN provided support and services. HX, YGZ, and YS reviewed and provided critical comments on the manuscript. YGZ and YS were obtained the funding. All authors read and approved the final manuscript.
